# Potential determinants of vaccine hesitancy among celiac disease patients: a single cohort analysis

**DOI:** 10.3389/fpubh.2023.1061617

**Published:** 2023-08-08

**Authors:** Shazia Rehman, Erum Rehman, Ondrej Holy

**Affiliations:** ^1^Department of Psychiatry, National Clinical Research Center for Mental Disorders and National Center for Mental Disorders, The Second Xiangya Hospital of Central South University, Changsha, Hunan, China; ^2^Mental Health Institute of Central South University, China National Technology Institute on Mental Disorders, Hunan Technology Institute of Psychiatry, Hunan Key Laboratory of Psychiatry and Mental Health, Hunan Medical Center for Mental Health, Changsha, Hunan, China; ^3^Department of Mathematics, Nazarbayev University, Nur-Sultan, Kazakhstan; ^4^Science and Research Centre, Faculty of Health Sciences, Palacký University Olomouc, Olomouc, Czechia

**Keywords:** celiac disease, vaccination hesitancy, Pakistan, policy, vaccine-preventable diseases, COVID-19

## Abstract

**Introduction:**

Though researchers and scholars have greatly emphasized addressing the influencing factors of vaccination hesitancy, little attention has been paid to patients with celiac disease. Addressing the variables hampering attitudes might help direct appropriate patient advocacy and doctor-patient communication endeavors to encourage vaccination among celiac disease patients. The present investigation seeks to explore the coverage against vaccine-preventable diseases, vaccination attitudes, and related possible factors among celiac disease patients in the Pakistani setting.

**Methods:**

A self-reported online survey was conducted in Islamabad, Pakistan, for celiac disease patients aged 18 and above. The questionnaire was completed by 226 participants, with a response rate of 43.8%. The influencing variables for vaccination hesitancy were examined, and 95% confidence intervals for the crude and adjusted odds ratios were computed.

**Results:**

Among the study population, the majority were females, with a ratio of 75.66%. A prominent proportion of 69.03% was observed for influenza vaccination, while 39.82% were unable to recall all of the vaccinations they had previously received. Only 7% of the patients were considered to have a negative attitude toward vaccination, compared to an estimated 76% who were in favor of it. The significantly positive influencing factors observed toward vaccination were being well-educated (graduate, master, or above), possible recurrence of vaccine-preventable diseases with declining vaccination coverage (adjusted OR: 13.36), and increased confidence in vaccines from health care experts compared to electronic media (*adjusted OR*: 8.41). Contrarily, practicing complementary and alternative medicines (*adjusted OR*: 5.59), willingness to get vaccinated again in the future (*adjusted OR*: 15.59), and prior negative perspectives (*adjusted OR*: 1.01) were the determinants with a significant negative association.

**Discussion:**

In conclusion, the outcomes of the current work raise the possibility that health practitioners may be accountable for inappropriately prescribing vaccines to this demographic since 77% of the participants had a favorable attitude toward vaccination. These findings could serve as a springboard for creating targeted immunization efforts to raise vaccination coverage against vaccine-preventive diseases among celiac disease patients.

## 1. Introduction

Presently, one of the most significant challenges of public health is vaccine hesitancy, which is a widespread, multifaceted, and constantly evolving phenomenon which may vary depending on the period, place, and vaccination type (1–3). The public discourse over vaccinations is a profoundly ingrained aspect of Western civilization and even worse in a low and middle-income setting like Pakistan (4, 5). Primary prevention has consistently been emphasized in Pakistan's healthcare infrastructure, and the country has had a strong legislative background of mandating immunizations since infancy (6, 7). As one of only two remaining polio-endemic nations, the country has previously seen significant resistance to polio vaccine programs (8, 9). As a result, any unfavorable attitudes against immunizations would jeopardize the entire endeavor.

**Figure 1:**
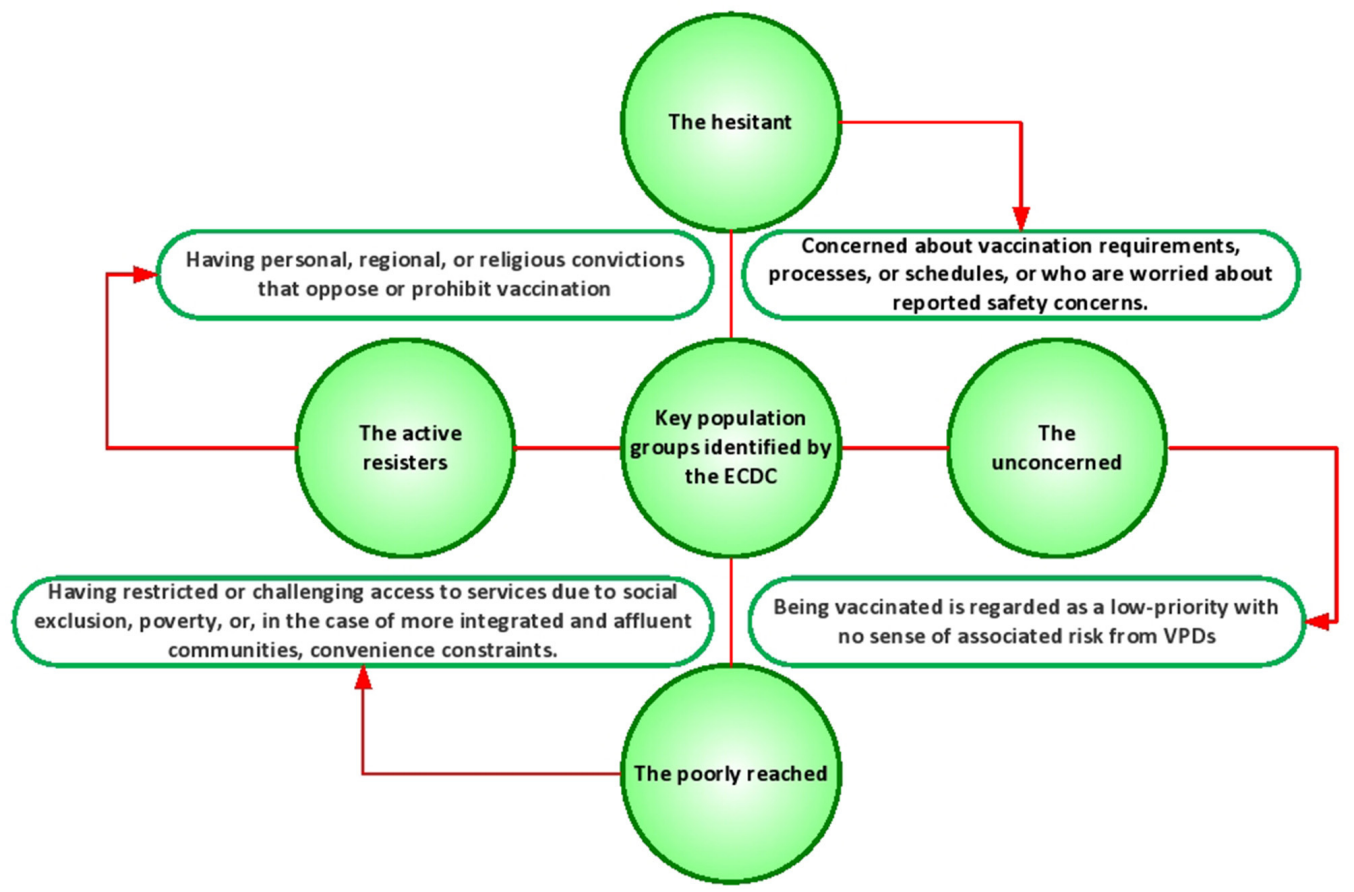
The key population groups identified by the ECDC.

Security concerns, unpleasant narratives, and personal experience are three significant drivers of vaccine hesitation. The reliability of the vaccine is a critical concern even for individuals who are very motivated to get a coronavirus disease 19 (COVID-19) vaccination (10–12). The possible role of COVID-19 in stimulating autoimmune responses has generated considerable interest. Consequently, there is an imperative to explore whether the administration of COVID-19 vaccines elicits the production of autoantibodies and subsequent development of autoimmune conditions. People are concerned about how rapidly a vaccine would have been developed and the fact that healthcare professionals and pharmaceutical corporations would not have known about all of the potential negative effects (13, 14). Vaccine hesitancy is frequently triggered by misguided thoughts about health, illnesses, and immunizations, that could have been affected by disinformation. The emphasis on the possible negative effects of vaccinations in the electronic media has resulted in waves of misunderstanding about vaccine safety, primarily regarding persistent adverse effects, the toxic effects of auxiliary and preservatives, and the immune system weakening (15, 16). Research commissioned by the European Center for Disease Control and Prevention (ECDC) in 2000 presented a first attempt to categorize this multifaceted scenario by separating patients into diverse factions (Figure 1) (17).

A revival of this concern has been attributed to the COVID-19 outbreak, the current global vaccination crusade, and the ensuing distribution of false documentation by anti-vaccination forces (11, 18). The older population with chronic illnesses including diabetes mellitus, hypertension, chronic renal disease (CRD), and chronic obstructive pulmonary disease (COPD) are more susceptible to infection, thus they are frequently urged to get immunized against vaccine-preventable diseases (VPDs) (19, 20). Older patients in Pakistan who participated in a recent post-pandemic survey had not shown a satisfactory response to the preventative measures provided by vaccinations (21). The potency of a booster shot in conferring substantial protection against serious illness and hospital admissions even among susceptible patients is also highlighted by research from Italy (22). However celiac disease, one of the most prevalent chronic autoimmune ailments with a distinctive histological and serological description brought on by consuming gluten in people with a genetic predisposition, frequently strikes young patients. Patients with celiac disease should typically be urged to take all basic vaccinations against VPDs as the overall populace does, even if it is unknown whether they have a usually higher chance of viral infections (23). In addition, several infectious agents might potentially affect patients with celiac disease. The European Society for the Study of Coeliac Disease (ESsCD) believes that there is a possibility that celiac disease is accompanied by hyposplenism or functional asplenia, which might decrease immunity to encapsulated bacteria and raise the chance of contracting such pathogens (24). As a result, celiac disease patients who are hyposplenic must be vaccinated against pneumococcus.

Doctors typically underestimate celiac disease owing to its irregular clinical manifestations and indications. Most celiac disease patients in Pakistan do not go to hospitals because of their peculiar indications. Several medical facilities in Pakistan, including ours, have seen an upsurge in the number of patients with celiac disease (25–29). According to the findings of these investigations, Pakistan is substantially more affected by celiac disease than formerly believed. The results of population screening initiatives now demonstrate that celiac disease is underdiagnosed and inadequately treated, and constitutes a more substantial challenge to public health in this region. Infectious diseases, as well as the possibility of vaccination to combat them, are controversial subjects not merely within the scientific world, but also in public discourse and attitude (15). With the strength of its impact, social media has helped to mainstream not only vaccination advocacy but also healthcare misconceptions, resulting in rising vaccine hesitation over the last decades (30). Vaccination hesitancy is described as “a delay in acceptance or refusal of vaccines despite availability of vaccination services” by the World Health Organization (WHO) Strategic Advisory Group of Experts on Immunization (SAGE) (31). It highlights a key challenge that has been thoroughly investigated to enhance patient-doctor communication in supporting preventive strategies and fostering vaccination acceptance, commencing with family doctors (32).

To the best of the author's knowledge, the research that is currently available provides scant evidence about whether celiac disease patients are more scared of vaccines than the general population or if they are informed of the immunizations they have already had or could be administered in the future. In response, it is imperative to ascertain the vaccination history and the attitude toward vaccination of celiac patients in the Pakistani populace. We sought to investigate the relationships between all the previous vaccinations history of celiac disease patients and their attitudes toward the vaccination so that potential variables could be identified. Addressing those variables hampering attitudes might help direct appropriate patient advocacy and doctor-patient communication endeavors to encourage vaccination among celiac disease patients.

## 2. Materials and methods

### 2.1. Data source and study population

The survey questionnaire (an anonymous web-based adapted version) was developed and sent via email to 514 celiac disease patients twice in 4 weeks by the Pakistan Institute of Medical Sciences, Islamabad, Pakistan in August 2021. Participants were requested to self-report their prior vaccination record as well as their opinions regarding vaccinations, which were categorized as either positive, negative, or neutral (using a multiple-choice assessment). The contributing variables for vaccinations were examined, and 95% confidence intervals for the crude and adjusted odds ratios (OR) were determined. The questionnaire was developed and verified by two biostatisticians, in the second round. Participants were not compensated with any privileges or finances as payment for answering the online survey. Three aspects were assessed by the online questionnaire which included social-demographic/occupational/behavioral details, information on celiac disease, and opinion/attitude toward vaccination. The adapted questionnaire was divided into seven sections and have been added in the Supplementary material.

### 2.2. Ethical considerations

The Ethics Committee of the PIMS Hospital in Islamabad, Pakistan, authorized this research (Approval Reference: KIIT-2021/0483). All protocols were carried out in compliance with the guidelines established by our institution's Ethics Committee and the Declaration of Helsinki's principles. Interviewees' involvement in the survey was regarded as informed consent. To protect the confidentiality of the participants, we did not request separate written informed consent. All participants' identities were maintained anonymously.

### 2.3. Statistical analysis

For categorical factors, absolute and relative frequencies were determined, while quantitative variables were summarized by averages and ranges. The study sample was computed based on the percentage of people who were projected to have a positive or negative attitude toward vaccination. A multivariable backward stepwise model for logistic regression comprised all factors identified in the univariate investigation to have a statistically significant correlation with vaccination attitude. To ensure a more conservative perspective, the multivariable analysis only considered variables with a *p* ≤ 0.20. In the logistic regression model, we also computed the crude and adjusted odds ratio with 95% confidence intervals. The significant threshold for regression analysis was 0.01 with a two-tailed test. With SAS version 9.3 (SAS Institute Inc., Cary, NC, USA), the overall analysis was performed.

## 3. Results

For the present investigation, 226 participants responded to the questionnaire yielding a response rate of ~43.8%. Table 1 highlights the baseline attributes of the study population with social demographic, occupational, and behavioral states. Among 226 participants, 171 (75.66%) were female patients. The interquartile range for age was 32–59 years, with 42 being the mean age. In addition, 60.18% of the study's participants were graduates, and celiac disease was suffered by every single patient (100%). It was interesting to note that among the selected sample, 93.81% of the participants had comorbid conditions (self-reported). 60.18% of the participants were diagnosed more than 10 years ago, while only 9.29% worked as healthcare providers. In the selected population, an estimated 10.18% were alcoholics and 23% were smokers. Around 13.27% of participants were vegetarians, and nearly 11% had a history of practicing complementary or alternative medicine.

**Table 1:** Basic social-demographic/occupational/behavioral attributes of the study population.

**Characteristics**	**Sample size (*n* %)**	**95% Confidence interval**
**Sex**		
Male	55 (24.35%)	19.76–28.26
Female	171 (75.66%)	70.78–79.15
**Age (years)**		
Mean (range)	42 (32–59%)	
**Marital status**		
Married	163 (72.12%)	69.11–75.94
Divorced/widowed	42 (18.58%)	15.53–21.19
Unmarried	21 (9.29%)	6.87–13.07
**Educational attainment**		
Undergraduate	45 (19.91%)	16.32
Graduate	136 (60.18%)	52.39
Masters and above	45 (19.91%)	16.72
**No. of family members**		
≤ 2	24 (10.62%)	5.98
>2	201 (88.94%)	78.15
**Disease**		
Celiac	226 (100%)	
**Healthcare worker**		
Yes	21 (9.29%)	5.91
No	205 (90.71%)	76.18
**Profession**		
Manager/entrepreneur/freelancer	53 (23.45%)	17.65
Employee/technical work	130 (57.52%)	47.19
Manual/crafting work	11 (4.87%)	1.17
Student/older adults/unemployed/housewife	32 (14.16%)	8.76
**Self-reported comorbidities**		
Yes	212 (93.81%)	68.15
No	14 (6.19%)	3.17
**Alcoholic**		
Yes	23 (10.18%)	7.15
No	203 (89.82%)	71.16
**Smoking**		
Yes	52 (23.00%)	18.55
No	174 (76.99%)	59.13
**Self-reported active lifestyle**		
Yes	132 (58.41%)	39.76
No	94 (41.59%)	30.16
**Vegetarian**		
Yes	30 (13.27%)	9.64
No	196 (86.72%)	71.19
**Practicing complementary or alternative medication (before/currently)**		
Yes	25 (11.06%)	8.80
No	201 (88.94%)	73.19
**No. of years since diagnosed**		
< 5	42 (18.58%)	14.78
5–10	48 (12.24%)	10.01
≥10	136 (60.18%)	51.09

The estimated vaccination histories for the study population are summarized in Table 2. Among 226 patients, a proportion of 69.03% of the sample's participants had received an influenza vaccination. In contrast, 40.71, 38.94, and 23.5% had received MMR, dengue, and polio vaccinations, respectively. In aggregate, only 38 completed their vaccination against tetanus, 25 received their meningitis vaccination, and 17 received their pneumococcus vaccination. In addition, almost 93 patients (39.82%) could not remember all their previous vaccinations received.

**Table 2:** Vaccination history of the study population with celiac disease.

**Vaccination history**	***n* (%)**	**95% Confidence interval**
Influenza	156 (69.03%)	61.11–70.78
Measles, mumps, and rubella (MMR)	92 (40.71%)	38.76–43.94
Dengue	88 (38.94%)	32.78–41.43
Polio	52 (23.00%)	19.56–24.08
Tetanus	38 (16.81%)	12.70–19.44
Meningitis	25 (11.06%)	8.89–13.66
Pneumococcus	17 (7.52%)	5.01–8.98
Participants unable to remember previous vaccination record	90 (39.82%)	31.90–44.62

Table 3 summarizes the statistics on attitudes toward vaccination for the celiac disease patients chosen for the present investigation. Only 7% of the patients were considered to have a negative attitude toward vaccination, compared to an estimated 76% who were in favor of it. However, barely 45 patients (19.91%) expressed a neutral response. One hundred seventy-six patients (77.88%) indicated a willingness for future vaccinations, and a comparable proportion indicated a willingness to get their children vaccinated in the future. Only 10 of them stated that they would not get their children immunized in the future, while 17% of those who responded indicated that they were only somewhat willing.

**Table 3:** Attitude toward vaccination in the study population.

	***n* (%)**	**95% Confidence interval**
**Attitude toward the vaccination**		
Positive	174 (76.99%)	70.01–82.56
Negative	7 (3.1%)	1.81–6.39
Neutral	45 (19.91%)	13.02–28.73
**Willingness to get vaccinated again in the future**		
Yes	176 (77.88%)	69.13–86.99
No	50 (22.12%)	19.11–33.32
**Willingness to get your children vaccinated in the future**		
Yes	176 (77.88%)	72.73–80.54
No	10 (4.42%)	1.12–5.00
Neutral	40 (17.70%)	14.07–20.25
**Considering the possible recurrence of VPDs in light of declining vaccination coverage**		
Yes	205 (90.71%)	86.99–93.14
No	21 (9.29%)	4.45–11.99
**The best strategy to prevent VPDs**		
Vaccination	57 (25.22%)	19.20–29.73
Vaccination + other preventive strategies	165 (73.00%)	70.16–77.64
Other (diet, physical activity, homeopathy, etc.)	4 (1.77%)	0.26–2.93
**Celiac disease/ongoing therapy as motivation for previous vaccination done**		
Agreed	21 (9.29%)	5.63–13.38
Not agreed	205 (90.71%)	87.11–94.35
**The prior negative perspective of vaccination (personally/family members/relatives reported/referred)**		
Yes	34 (15.04%)	10.19–19.43
No	192 (84.96%)	79.15–86.01
**Increased confidence in vaccines from health care experts compared to electronic media**		
Yes	220 (97.35%)	90.90–99.18
No	6 (2.65%)	0.79–3.10

The majority of 226 patients (90.71%) considered that the decline in vaccine coverage may lead to a potential resurgence of VPDs, and 222 stated that vaccinations alone (25.22%) or in conjunction with other preventative measures (673%) were the best strategy to avoid VPDs. In contrast, there appeared a negligible ratio that had a conflict. Out of 226 patients, 205 indicated that celiac disease had not been the rationale for prior vaccinations. Only 34 patients (15.04%) self-reported having had a negative vaccination experience in the past. Moreover, 97.35% of the patients demonstrated increased confidence in the information on vaccines offered by healthcare experts than in the information provided by the electronic media.

**Figure 2:**
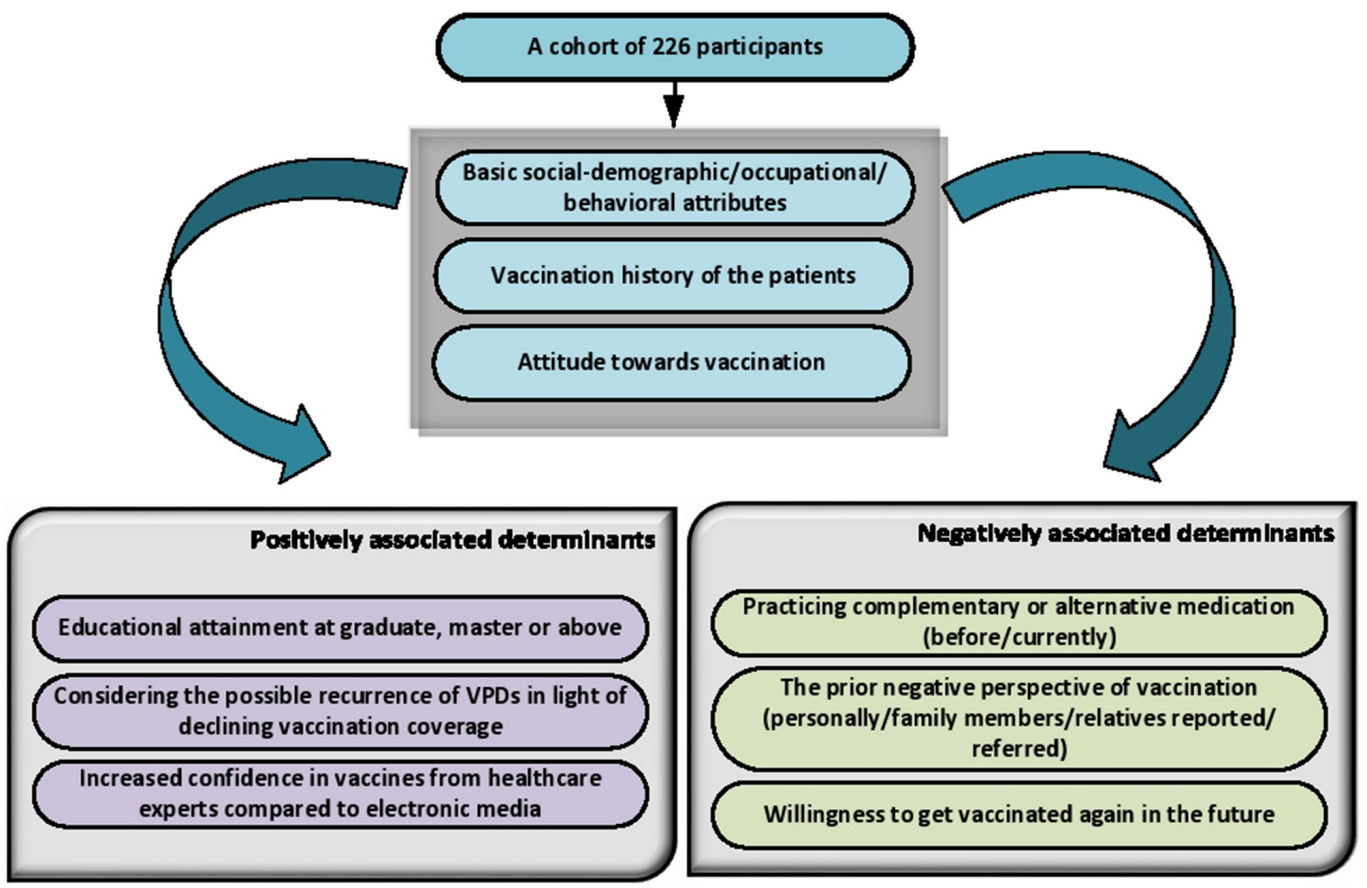
Potential factors influencing the attitude of celiac disease patients toward vaccination.

The outcomes of the univariate and multivariate analyses are summarized in Table 4. Educational attainment at a graduate level (*adjusted OR*: 6.45 with *p* < *0.000*) and master or above level (*adjusted OR*: 11.01 with *p* < *0.000*) were the characteristics that positively and significantly influenced the attitude of the patients toward vaccination. Additionally, taking into account the possible recurrence of VPDs with declining vaccination coverage rates (*adjusted OR*: 13.36 with *p* < *0.000*) and increased confidence in vaccines from healthcare experts compared to mass media (*adjusted OR*: 8.41 with *p* < *0.000*) shown to have a significant positive impact on the attitude of celiac disease patients toward vaccination. Contrarily, it was found that patients' attitudes toward vaccination were negatively impacted by their use of complementary and alternative therapies (*adjusted OR*: 5.59 with *p* < *0.001*), their willingness to get vaccinated again in the future (*adjusted OR*: 15.59 with *p* < *0.000*), and their prior negative perspectives (*adjusted OR*: 1.01 with *p* < *0.000*). The potential determinants with positive and negative influences on vaccination hesitancy can be presented in graphical form in Figure 2.

**Table 4:** Univariate and multivariate analyses of the selected variable influencing the attitude toward vaccination for patients with celiac disease.

	**Crude OR (95% CI)**	***P-*value**	**Adjusted OR (95% CI)**	***P-*value**
**Sex**				
Male	Ref			
Female	1.32 (0.54–1.09)	0.23		
**Age (years)**	1.09 (0.85–2.11)	0.50		
**Marital status**				
Married	2.81 (0.96–3.33)	0.11		
Divorced/widowed	0.78 (0.23–1.65)	0.53		
Unmarried	Ref			
**Educational attainment**				
Undergraduate	Ref		Ref	
Graduate	3.88 (1.66–5.43)	**0.001**	6.45 (4.94–12.10)	**0.000**
Masters and above	7.19 (5.06–10.72)	**0.000**	11.01 (9.52–22.39)	**0.000**
**Adherence to a gluten-free diet**				
Yes	5.87 (3.99–12.30)	0.71		
No	Ref			
**Healthcare worker**				
Yes	0.41 (0.11–2.63)	0.12		
No	Ref			
**Smoking**				
Yes	0.98 (0.55–2.09)	0.64		
No	Ref			
**Self-reported physical activity**				
Yes	4.33 (2.61–6.04)	0.82		
No	Ref			
**Alcoholic**				
Yes	2.74 (1.50–5.88)	0.75		
No	Ref			
**Practicing complementary or alternative medication (before/currently)**				
Yes	8.43 (5.09–20.64)	**0.000**	5.59 (3.28–8.41)	**0.001**
No	Ref			
**Adherence to other preventive measures**				
Yes	0.59 (0.09–2.28)	0.35		
No	Ref			
**Willingness to get vaccinated again in the future**				
Yes	18.61 (16.16–31.74)	**0.000**	15.59 (11.04–20.36)	**0.000**
No	Ref			
**Considering the possible recurrence of VPDs in light of declining vaccination coverage**				
Yes	9.92 (6.71–16.28)	**0.000**	13.36 (9.98–17.05)	**0.000**
No	Ref		Ref	
**The prior negative perspective of vaccination (personally/family members/relatives reported/referred)**				
Yes	1.23 (0.95–4.73)	**0.000**	1.01 (0.53–2.19)	**0.000**
No	Ref		Ref	
**Increased confidence in vaccines from healthcare experts compared to electronic media**				
Yes	5.39 (3.39–8.64)	**0.001**	8.41 (6.93–12.05)	**0.000**
No	Ref		Ref	

Bold shows significant p-values (P < 0.01).

## 4. Discussion

To the best of the author's knowledge, the present survey is a first attempt to document vaccinations and attitudes toward vaccinations among celiac disease patients in the Pakistani community. Statistics demonstrate that nearly 40% of celiac disease patients could recall their vaccination history. The most reported vaccination received is against influenza considering that this vaccination has been mandatory in the Pakistani population since childhood. The self-reported low vaccination rates of the research participants are probably an underestimation of their actual vaccination status. This is corroborated by the fact that 39.8% of the participants had trouble recalling past immunizations, which alludes to a lack of emphasis on the significance of primary prevention and underscores the necessity for effective patient-doctor communication. Very few patients reported receiving a vaccination against encapsulated microbes, which is even more pertinent. Presently, data are scarce on the actual proportion of celiac disease patients who have had a pneumococcal vaccine; however, this suggests that this vaccination is significantly neglected in adult celiac disease patients (33). Our findings are consistent with a prior study conducted in 2013 that examined 119 celiac disease patients 65 years of age or older with at least one comorbid condition and discovered that only 19.2% of the sample patients had been vaccinated against pneumococcus, indicating that the accurate vaccination status in the celiac disease population has not evolved dramatically in the past years (34).

According to prior evidence, the incidence of hyposplenism in celiac disease patients ranges from 19% in cases of simple celiac disease without an autoimmune illness to 80% in settings when premalignant or malignant lesions are evident. Likewise, the frequency of splenic hypofunction rises in people with celiac disease who are also diagnosed with autoimmune diseases (e.g., autoimmune thyroiditis or insulin-dependent diabetes). Splenic hypofunction is unrelated to the period of a gluten-free diet (35, 36). This relationship is significant because hyposplenic individuals are at higher risk of developing catastrophic inflammation caused by gram-negative bacteria (e.g., capnocytophaga canimorsus) as well as encapsulated bacteria (e.g., *Streptococcus pneumonia, Neisseria meningitides*, and Haemophilus influenza type b) (37, 38). In a survey conducted among general practitioners in Pakistan, their level of understanding regarding the differentiation between celiac disease and irritable bowel syndrome was investigated. Results indicated a tendency to misdiagnose celiac disease as irritable bowel syndrome. The challenges associated with the diagnosis of celiac disease arise primarily from the limited awareness surrounding the condition and the variability of its symptoms. These factors collectively hinder timely identification and subsequent treatment of affected individuals (39).

An abundance of investigations has been conducted so far, particularly pneumococcal vaccination, owing to the potentially catastrophic illnesses linked to encapsulated bacteria in celiac patients. The pneumococcal vaccination should indeed be taken into consideration for patients with celiac disease, paying great emphasis to those who are 15–64 years old and have never experienced a vaccination before since celiac disease is significantly correlated to an elevated risk of *Streptococcus pneumoniae* (*S. pneumoniae*) infection, according to Simons et al. (40). Evidence also suggests that giving pneumococcus vaccinations to people with celiac disease who are older at the time of diagnosis, have concurrent autoimmune ailments, have complex celiac disease, have experienced substantial infections or sepsis in the past, or who have venous thromboembolism (VTE) and atrophic spleen (41). The imperative of strengthening effective patient-doctor communication is emphasized by the possible significant risk of inflammation in celiac disease patients associated with hyposplenism (41, 42).

Despite one out of five patients exhibiting a somewhat undesirable opinion, the outcomes of the present work indicate a relatively significantly positive attitude toward vaccinations, with an intent of getting vaccinated in the future (against any infectious disease), also demonstrated a significant attitude toward vaccinations. Intriguingly, the majority of the participants claimed that their celiac ailment had not encouraged them to get their previous vaccines, indicating that they did not perceive a risk of developing an infectious disease as a result of their celiac condition.

This is also very significant since celiac disease is a chronic autoimmune condition that, in rare instances, might necessitate immunosuppressive medication, increasing the risk of contracting infectious illnesses (43, 44). Furthermore, it is crucial to note that 90% of participants believe that the decline in vaccine coverage would lead to a potential resurgence of VPDs. Contrary to a reduced rate of vaccinations that resulted from our research, exhibited a considerable positive relationship with vaccination attitude, suggesting the understanding of the significance of vaccination programs.

As previously documented, a positive attitude toward the use of any other drug has a strong sign, and a negative relationship with the attitude toward vaccinations (45, 46). Considering any other drugs patients who believe that vaccinations and other pharmaceuticals regularly administered by health professionals are hazardous may seek alternative treatments and techniques such as chiropractic and acupuncture (especially in a setting with scarce evidence) (47). The majority of participants believe healthcare providers are more than the media for health-related information. Effective communication may be used to increase immunization rates among celiac disease patients, which is essential for public awareness initiatives. The responsibility of general clinicians is particularly critical since they are frequently the initial referral physician for celiac disease patients. In essence, they should assure that celiac disease patients are adequately vaccinated, if appropriate, against encapsulated microbes (48, 49). Besides that, considerable research, attention, and endeavors should be put into persuading patients who are not well educated and unaware of the adverse outcomes of the undergoing disease. Healthcare professionals should also investigate these reports to gauge the exact spectrum of reported adverse vaccination-related experiences and to establish whether they were legitimate side effects or merely placebo effects. We discovered that their opinions regarding vaccinations were considerably impacted by these unfavorable experiences.

In light of the preceding research and our present findings, we may recommend certain measures that may be adapted to ramp up vaccination acceptance among patients with celiac disease by implementing specific practices. These suggestions may include strengthening the engagement of healthcare experts who routinely handle people with celiac disease, encouraging policies that offer comprehensive guidance on how to effectively advocate for vaccination, and dissemination of the immunization message to all patients with the assistance of patient associations, resulting in the expansion of the immunization culture, improved vaccination practices for celiac disease patients using awareness programs as well as the delivery of vaccinations within of vaccination regimens, and enhancing the doctor-patient relationship for those who are more likely to be hesitant about vaccinations (50–54).

The best approach to ramping up vaccination coverage among celiac disease patients is through education. Inappropriate conduct that violates the public health guidelines suggested for both the general populace and at-risk groups, can emerge from the disparity between the accurate and the perceived risk of VPDs. Although there is an elevated level of awareness, it is crucial to receive the necessary precautions and directions from medical professionals. The use of digital technologies for improving vaccination initiatives might have yielded significant benefits (55).

## 5. Conclusion

In conclusion, the outcomes of the current work raise the possibility that health practitioners may be accountable for inappropriately prescribing vaccines to this demographic since 77% of the participants had a favorable attitude toward vaccination. These findings could serve as a springboard for creating targeted immunization efforts to raise vaccination coverage against VPDs among celiac disease patients. Healthcare professionals should inquire about how well-vaccinated celiac disease patients are, and counsel them to acquire all recommended vaccinations as well as any additional, potentially life-threatening vaccinations. To strengthen patient communication and design targeted vaccination campaigns for vaccine-hesitant patients, it may be advantageous to identify the factors that influence patients' attitudes regarding vaccinations.

The authors assert that it is imperative for the scientific community and public health officials to ascertain the factors and determinants that influence celiac disease patients' inclination toward vaccines. This understanding is crucial in order to devise tailored vaccination initiatives and optimize communication between patients and doctors.

## Data availability statement

The raw data supporting the conclusions of this article will be made available by the authors, without undue reservation.

## Ethics statement

The studies involving human participants were reviewed and approved by the Ethics Committee of the PIMS Hospital in Islamabad, Pakistan, authorized this research (Approval Reference: KIIT-2021/0483). All protocols were carried out in compliance with the guidelines established by our institution's Ethics Committee and the Declaration of Helsinki's principles. Interviewees' involvement in the survey was regarded as informed consent. To protect the confidentiality of the participants, we did not request a separate written informed consent. All participants' identities were maintained anonymous. Written informed consent for participation was not required for this study in accordance with the national legislation and the institutional requirements.

## Author contributions

All authors listed have made a substantial, direct, and intellectual contribution to the work and approved it for publication.
