# Supplementary material for: Potential determinants of vaccine hesitancy among celiac disease patients: a single cohort analysis
Source: Front Public Health. 2023 Aug 8;11:1061617. doi: 10.3389/fpubh.2023.1061617 (PMC10442556; doi:10.3389/fpubh.2023.1061617)
Supplement: Supplementary file 1 [file Table_1.DOCX]

Sections and sub-sections of the vaccination questionnaire

| ***Section-I*** | Social-demographic information (sex, age, educational attainment, marital status, family status, and professional status especially if the healthcare provider |
| --- | --- |
| ***Section-II*** | Information regarding the course of celiac disease in terms of classification, disease duration, therapies, and adherence to the gluten-free diet. |
| ***Section-III*** | Lifestyle, health-related behaviors, and attitudes (smoking, physical activities, and approach to screening services) |
| ***Section-IV*** | Knowledge and perceptions regarding vaccination and vaccine-preventable diseases |
| ***Section-V*** | Vaccination history |
| ***Section-VI*** | Source of information about the vaccine (healthcare experts, electronic media, or pharmacists) |
| ***Section-VII*** | Prior negative perspectives of vaccination (personally/family members/relatives reported/referred) |
